# Exploding Head Syndrome Accompanied by Repeating Panic Attacks: A Case Report

**DOI:** 10.3389/fpsyt.2020.613420

**Published:** 2021-01-22

**Authors:** Yoshiyuki Kaneko, Akiomi Kawae, Kaori Saitoh, Yasuhiro Gon, Makoto Uchiyama, Masahiro Suzuki

**Affiliations:** ^1^Department of Psychiatry, Nihon University School of Medicine, Tokyo, Japan; ^2^Sleep Medicine Center, Nihon University Itabashi Hospital, Tokyo, Japan; ^3^Division of Respiratory Medicine, Department of Internal Medicine, Nihon University School of Medicine, Tokyo, Japan; ^4^Tokyoadachi Hospital, Tokyo, Japan

**Keywords:** exploding head syndrome, sleep disorder, parasomnia, panic attack, clonazepam, case report

## Abstract

To the best of our knowledge, we report here for the first time a case of exploding head syndrome (EHS) that caused repeating panic attacks. A 62-year-old woman experienced a sudden sensation of a loud noise just before going to sleep. The frequency of these episodes rapidly increased to multiple times per night, and she soon began to fear sleep, which led to the occurrence of nighttime panic attacks. She was diagnosed with EHS at our sleep clinic, and clonazepam was prescribed accompanied by reassurance about the benign nature of this syndrome. The intensity of the loud noise gradually reduced, and her fear of sleep and panic attacks disappeared at around the same time. In this report, we argue the importance of gaining further knowledge about EHS, including that about complicating psychiatric symptoms and that about its treatment.

## Introduction

Exploding head syndrome (EHS) is a parasomnia characterized by episodes in which loud noises or explosions are perceived when going to sleep or awakening (1). Although only a small number of cases of EHS leading to psychiatric complications have been reported (2), episodes of EHS often cause fear and distress in affected patients (1). Here, we present a case of EHS as a suggested cause of repeating panic attacks. To the best of our knowledge, this is the first time a case of EHS leading to panic attacks has been reported. Additionally, we briefly discuss the importance of gaining further knowledge about this syndrome and its treatment.

## Case Description

**Figure 1:**
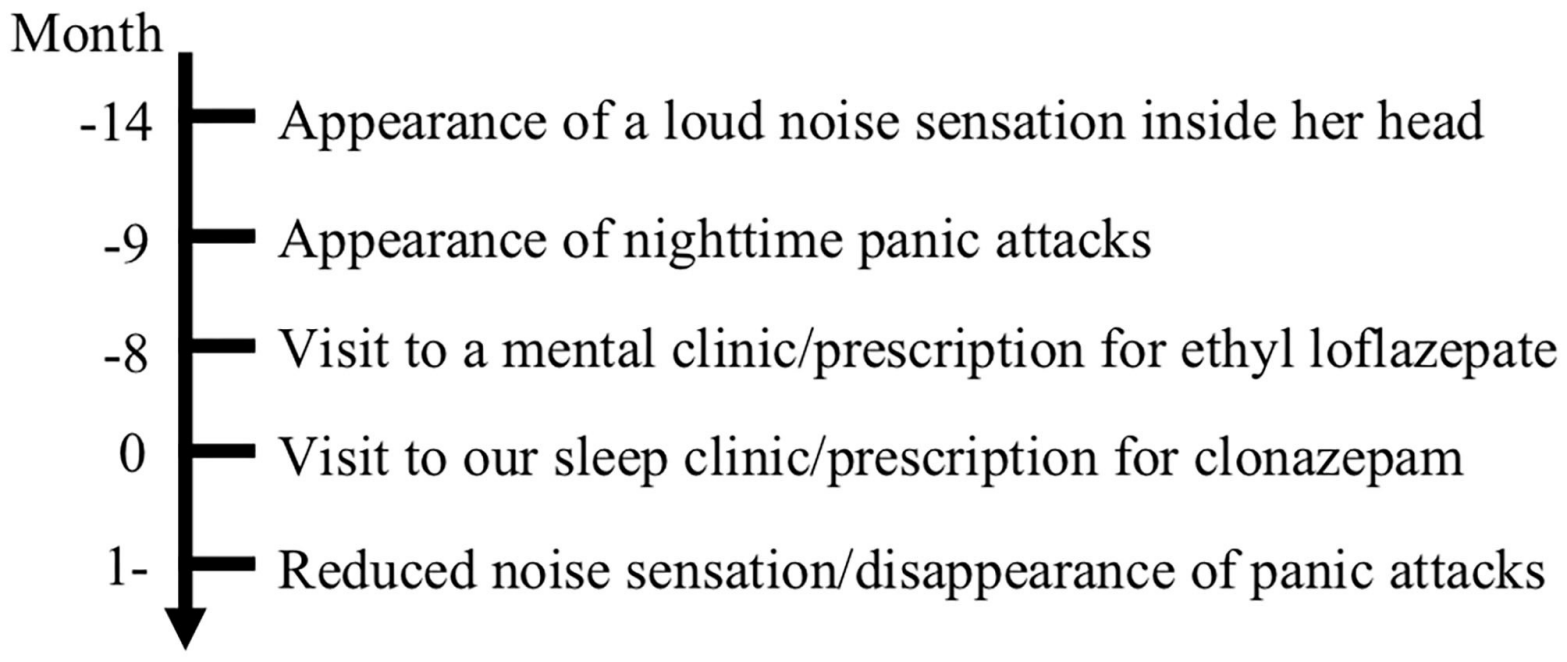
Case history timeline.

A 62-year-old woman was admitted to the clinic at Nihon University Itabashi Hospital Sleep Medicine Center to investigate a 14-month history of a sudden sensation of a loud noise inside her head (Figure 1). She described the noise as the sound of a guitar string and honking bus horn, and only experienced it when going to sleep. The noise sensation was not accompanied by pain. Initially, she experienced these episodes less than once per month; however, the frequency rapidly increased to more than once a night, but the cause of this increase was unknown. She developed a fear that the loud noise would occur again when going to bed, which caused difficulty falling asleep. Subsequently, she began to experience frequent nighttime panic attacks accompanied by shortness of breath as a main symptom. She finally decided to call an ambulance and visited the emergency room repeatedly. She was referred to a mental clinic, where she was diagnosed as having panic attacks based on the International Classification of Diseases, 10th revision, and prescribed ethyl loflazepate for 3 months, but this did not stop the loud noise sensation. She then visited our sleep clinic, where she described having a clear recollection of the episodes and an intense fear that she must have a serious brain disease. She had no medical or psychiatric history except for ureteral stones, no family history of psychiatric disorders, no psychosocial history, and no psychiatric symptoms except for panic attacks and a fear of sleep. She had no complaints of headaches or nightmares, no abnormal findings in a physical examination, including neurological, cardiovascular, and respiratory examinations, and no abnormal findings in laboratory examinations, including a blood test, C-reactive protein and liver function, and renal and thyroid functions. Moreover, no abnormal findings were observed in T1-weighted, T2-weighted, fluid-attenuated inversion recovery, or diffusion-weighted magnetic resonance images obtained using a 1.5 T scanner. She did show symptoms of insomnia, especially poor subjective sleep quality, based on the Japanese version of the Athens Insomnia Scale (3), with a score of 10 points [range: 0–24 points, a cutoff score ≥8 indicates insomnia (4)]. Polysomnography (PSG) showed no evidence of sleep disorders, and electroencephalography recorded during PSG showed no epileptiform activity, while she experienced a horn noise when going to sleep. Subsequently, she was diagnosed with EHS, and patient education was provided with reassurance about the benign nature of the syndrome. In addition, clonazepam 0.25 mg was prescribed to help reduce her loud noise sensations and alleviate her panic attacks. She was adequately given information about the treatment she received, and she shared their perspective on the treatment. She adhered to her medication without fail, and no adverse effect was observed. The intensity of the noise sensations gradually decreased, and her panic attacks and fear of sleep disappeared at around the same time. After 6 months of treatment with no change of clonazepam dose, the frequency of episodes decreased to once a week. We obtained informed written consent from the patient authorizing publication of clinical case.

## Discussion

Here, we described a patient with EHS who was repeatedly admitted to the emergency room because of panic attacks and anticipatory anxiety for the sensation of a loud noise. In this case, it took more than 1 year for the patient to be admitted to our sleep clinic and receive a diagnosis of EHS. Although EHS has a benign prognosis (5, 6), the phenomenon is often frightening to those who believe that it is caused by a problem in the brain (7). Since the symptoms of EHS are often relieved only by patient education and reassurance about the benign nature of the syndrome (7–9), early detection is beneficial for affected patient. EHS is not well-known among most clinicians, even though it has been reported to have a high lifetime prevalence of 30–40% (10). Therefore, knowledge about this syndrome, including that about complicating psychiatric symptoms, and its treatment should be shared widely between clinicians.

In this case, we prescribed a small amount of clonazepam, a benzodiazepine, because it is useful for preventing panic attacks, probably because of its serotonergic properties (11), as well as its GABAergic properties. In addition to preventing panic attacks, clonazepam may have decreased the frequency of EHS episodes and the intensity of the loud noise. EHS has been reported to be ameliorated by clomipramine (8), amitriptyline (12), and duloxetine (2), all of which have serotonergic properties, similar to clonazepam. The common characteristics of these agents may suggest that the symptoms of EHS can be suppressed via the serotonin system. However, since EHS can be relieved only through patient education and reassurance (7–9), the clinical effects of clonazepam on EHS should be confirmed in further case-control studies.

## Data Availability Statement

The original contributions presented in the study are included in the article/Supplementary Material, further inquiries can be directed to the corresponding author/s.

## Ethics Statement

Ethical review and approval was not required for the study on human participants in accordance with the local legislation and institutional requirements. The patients/participants provided their written informed consent to participate in this study. Written informed consent was obtained from the individual(s) for the publication of any potentially identifiable images or data included in this article.

## Author Contributions

YK, AK, MU, and MS contributed to the conception and design of the study. AK, KS, YG, and MS acquired the data. YK and AK wrote the first draft of the manuscript. YK and MS wrote sections of the manuscript. All authors contributed to revising the manuscript and read and approved the final version to be submitted.

## Conflict of Interest

The authors declare that the research was conducted in the absence of any commercial or financial relationships that could be construed as a potential conflict of interest.
