# Supplementary material for: Exploding Head Syndrome Accompanied by Repeating Panic Attacks: A Case Report
Source: Front Psychiatry. 2021 Jan 22;11:613420. doi: 10.3389/fpsyt.2020.613420 (PMC7862109; doi:10.3389/fpsyt.2020.613420)
Supplement: Supplementary file 1 [file Data_Sheet_1.PDF]

CARE case report guidelines

CARE Checklist of information to include when writing a case report

(cc) BY-NC-ND

✓

Topic	Item	Checklist item description	Reported on Line
Title	1	The diagnosis or intervention of primary focus followed by the words "case report"	1 – 2
Key Words	2	2 to 5 key words that identify diagnoses or interventions in this case report, including "case report"	13 – 14
Abstract (no references)	3a	Introduction: What is unique about this case and what does it add to the scientific literature?	17 – 18
	3b	Main symptoms and/or important clinical findings	18 – 23
	3c	The main diagnoses, therapeutic interventions, and outcomes	21 – 23
	3d	Conclusion—What is the main "take-away" lesson(s) from this case?	23 – 25
Introduction	4	One or two paragraphs summarizing why this case is unique (**may include references**)	28 – 32
Patient Information	5a	De-identified patient specific information.	36 – 67
	5b	Primary concerns and symptoms of the patient.	36 – 43
	5c	Medical, family, and psycho-social history including relevant genetic information	48 – 50
	5d	Relevant past interventions with outcomes	42 – 47
Clinical Findings	6	Describe significant physical examination (PE) and important clinical findings.	50 – 52
Timeline	7	Historical and current information from this episode of care organized as a timeline	
Diagnostic Assessment	8a	Diagnostic testing (such as PE, laboratory testing, imaging, surveys).	50 – 59
	8b	Diagnostic challenges (such as access to testing, financial, or cultural)	50 – 59
	8c	Diagnosis (including other diagnoses considered)	59 – 60
	8d	Prognosis (such as staging in oncology) where applicable	64 – 66
Therapeutic Intervention	9a	Types of therapeutic intervention (such as pharmacologic, surgical, preventive, self-care)	59 – 62
	9b	Administration of therapeutic intervention (such as dosage, strength, duration)	61 – 62
	9c	Changes in therapeutic intervention (with rationale)	N/A
Follow-up and Outcomes	10a	Clinician and patient-assessed outcomes (if available)	64 – 66
	10b	Important follow-up diagnostic and other test results	N/A
	10c	Intervention adherence and tolerability (How was this assessed?)	63 – 64
	10d	Adverse and unanticipated events	63 – 64
Discussion	11a	A scientific discussion of the strengths AND limitations associated with this case report	70 – 71, 86 – 88
	11b	Discussion of the relevant medical literature **with references**.	70 – 88
	11c	The scientific rationale for any conclusions (including assessment of possible causes)	72 – 77, 80 – 88
	11d	The primary "take-away" lessons of this case report (without references) in a one paragraph conclusion	77 – 79
Patient Perspective	12	The patient should share their perspective in one to two paragraphs on the treatment(s) they received	62 – 63
Informed Consent	13	Did the patient give informed consent? Please provide if requested	Yes ☑ No ☐
